# Spider Mite Response, Agronomic Performance, and Stability of a *Urochloa* spp. Diversity Panel Under Field Conditions

**DOI:** 10.3390/plants15071117

**Published:** 2026-04-05

**Authors:** Adrian Mating’i Kimani, David Kariuki Muruu, Paula Espitia-Buitrago, Sylvia Henga, Catherine Muui, Frank Chidawanyika, Rosa Noemi Jauregui

**Affiliations:** 1 International Center of Insect Physiology and Ecology (ICIPE), Nairobi P.O. Box 30772-00100, Kenya; kimaniadrian@gmail.com (A.M.K.); fchidawanyika@icipe.org (F.C.); 2 Department of Agricultural Science and Technology, School of Agriculture and Environmental Sciences, Kenyatta University, Nairobi P.O. Box 43844-00100, Kenya; 3 Tropical Forages Program, International Center for Tropical Agriculture (CIAT), Nairobi P.O. Box 823-00621, Kenya; d.muruu@cgiar.org; 4 Tropical Forages Program, International Center for Tropical Agriculture (CIAT), Palmira A.A. 6713, Colombia; 5 Department of Zoology and Entomology, University of the Free State, Bloemfontein P.O. Box 339, South Africa

**Keywords:** *Brachiaria*, biotic stress, AMMI, host-plant resistance, forage breeding, East Africa

## Abstract

Spider mites (*Oligonychus trichardti*) are emerging as a major constraint to *Urochloa* forage productivity in East Africa; however, knowledge of genotypic variation and tolerance remains limited. Herein, 55 *Urochloa* genotypes were evaluated under field-infested and non-infested conditions across two seasons using an alpha-lattice design. Agronomic and physiological traits, including plant height (PH), tiller number (TN), the Normalized Difference Vegetation Index (NDVI), total dry weight (TDW), and mite damage indices (visual severity index (VSI) and stress tolerance index (STI)) were assessed. Infestation reduced biomass by 22.4% on average, with reductions of up to 45% in susceptible genotypes. Significant genotypic variation was detected for PH, TN, TDW, and VSI. Heritability estimates under mite infestation were moderate to high for all traits except TDW, suggesting that direct selection of these traits could be effective in breeding programs aimed at improving mite resistance. VSI showed a strong negative correlation with NDVI (r = −0.63), supporting its value as a phenotyping indicator of spider mite response. Additive main effects and multiplicative interaction (AMMI) analysis revealed significant genotype × environment interactions for TDW. The AMMI biplot identified Xaraes, ILRI_13369, and ILRI_14787 as high-yielding and stable genotypes, while the AMMI Stability Value (ASV) and the Weighted Average of Absolute Scores from the Best Linear Unbiased Prediction (WAASB) identified CIAT_16122, CIAT_664, ILRI_14801, ILRI_14787, and ILRI_13266 as the most stable and broadly adapted across environments. STI further highlighted ILRI_13751 (2.71) and ILRI_13531 (2.58) as highly tolerant under stress. Overall, the study reveals substantial exploitable genetic diversity and identifies stable, high-yielding, and mite-tolerant genotypes suitable for breeding to improve *Urochloa* productivity in East Africa.

## 1. Introduction

*Urochloa* P.Beauv. (syn. *Brachiaria* (Trin.) Griseb) is a C4 perennial grass native to Eastern Africa’s tropical and subtropical regions, that grows in conditions characterized by mean temperatures of 18–27 °C and annual rainfall of 700–1500 mm [1]. As a key forage crop, it underpins both livestock production and rural economies [2]. Beyond climatic adaptability [3,4,5], *Urochloa* offers high-quality biomass that boosts milk yields (15–40%), and contributes to reducing greenhouse gas emissions [6,7].

Forage production is increasingly threatened by pests and diseases [8]. In the Americas, spittlebug damage prompted intensified breeding efforts in *Urochloa* spp. because of its major impact on forage productivity [9,10]. In Africa, spider mites (Acari: Tetranychidae) pose a similarly serious constraint, causing yield losses of up to 90% in East Africa [5,8,11]. The ILRI and CIAT genebanks maintain diverse *Urochloa* accessions, including genotypes that have recently been identified as moderately resistant to *Oligonychus trichardti* [5]. Host-plant resistance (HPR) offers a promising and sustainable strategy for managing spider mite damage through mechanisms such as tolerance, antibiosis, and antixenosis [12]. Nevertheless, field-based studies integrating agro-morphological traits, physiological indicators, and genetic variability to characterize mite resistance in *Urochloa* remain scarce. Breeding for resistance requires the combined evaluation of genetic parameters, including Phenotypic coefficient of Variation (PCV), Genotypic coefficient of Variation (GCV), and broad-sense heritability (H^2^) [13,14], together with physiological and stress-related indices such as the NDVI, VSI, and STI [15,16,17]. When assessed across multiple environments, these traits can be further interpreted using the AMMI model to disentangle genotype-by-environment interactions. Such approaches help distinguish broadly adapted from specifically adapted genotypes and support the identification of stable, pest-tolerant germplasm for the development of resilient, high-yielding forage cultivars [18,19].

Despite the agronomic importance of *Urochloa* in East Africa, integrated field studies combining mite damage phenotyping, agronomic performance, genetic variability, and multi-environment stability remain limited. In this study, we evaluated 55 *Urochloa* genotypes across two years under infested and non-infested field conditions using the visual severity index (VSI), stress tolerance index (STI), agro-morphological traits, and AMMI analysis of total dry weight (TDW). We hypothesized that: (i) genotype × environment interaction would significantly affect TDW under contrasting mite pressure, allowing discrimination between broadly adapted and specifically adapted genotypes; and (ii) a subset of genotypes would combine low mite damage with high biomass under infestation, making them promising candidates for breeding spider mite-tolerant cultivars. This study aims to support breeding decisions for resilient forage production in East Africa by integrating pest response and yield stability.

## 2. Materials and Methods

The panel comprised 55 genotypes: 10 accessions from CIAT’s Genebank, 7 hybrids from CIAT’s interspecific *Urochloa* (*U. ruziziensis* × *U. brizantha* × *U. decumbens*) breeding program, and 38 accessions from ILRI’s Genebank (Table 1).

The field study was conducted at the International Center of Insect Physiology and Ecology, Thomas Odhiambo Campus (ITOC), Mbita Point, Kenya (0°25′52.2″ S 34°12′29.0″ E), in 2023 and 2024. Mbita is a tropical region with an annual average temperature of 23.7 °C and an average rainfall of 1780 mm per year. The field site is 1100 m above sea level (m.a.s.l.) with vertisol soils.

Two adjacent but separately managed fields were used to represent contrasting pest conditions: Field A, artificially infested with spider mites, and Field B, a non-infested control. Each field was established as an alpha-lattice design, with six blocks in Field A and four blocks in Field B. The 55 genotypes were initially sown in sand trays in the greenhouse and then transplanted to the field in 1 m^2^ plots at 1.5 m × 1.5 m spacing. Both fields were surrounded by a continuous Napier grass (*Pennisetum purpureum* Schumach) border. Within 60 days after transplanting, two standardization cuts spaced one-month apart were performed, followed by top-dressing with CAN (27% Nitrogen) at a rate of 50 kg N ha^−1^, to promote plant growth and development. To establish spider mite pressure in Field A, a pure colony of *O. trichardti* was maintained on *Urochloa* hybrid cv. Mulato II. One-time artificial infestations were conducted in 2023 and 2024, three weeks after the second standardization cut. In this way, the vegetative stage concurred with the dry months occurring between June and September, which is the period of natural occurrence of spider mite infestation. Each plot in the infested field was inoculated using the same procedure: 30 adult female mites were transferred to a single excised leaf and placed in a leaf axil of the target plant. Applying a fixed number of mites per plot under the same crop stage was intended to standardize initial infestation pressure across genotypes. Mite infestation was verified before scoring for visual severity index (VSI) commenced. In Field B, abamectin (Dynamec 1.8 EC, 18 g/L emulsifiable concentrate, EC; Syngenta, Basel, Switzerland) was applied biweekly at a rate of 0.75 mL L^−1^ of water following the manufacturer’s recommendations to suppress spider mites, thereby maintaining the non-infested control treatment.

### 2.1. Data Collection

Plant height (PH), Normalized Difference Vegetation Index (NDVI), and leaf damage/visual severity index (VSI) data were collected weekly over six weeks in years 1 and 2; tiller number (TN) was collected biweekly during this same period. PH was measured from the soil to the average height of the plot, NDVI was measured using the Green Seeker Trimble tool by scanning each experimental unit for an average of 5–6 s [20], and TN was determined by counting individual stems in every plot. VSI was assessed by visually examining 2–3 fully developed leaves from both the upper and middle canopy affected leaves on each plant [5,21]. The presence and severity of yellowing, streaking, or anthocyanin was subsequently used to estimate the extent of chlorosis caused by mite feeding based on a visual severity damage scale (0–10) (Table 2).

The sampling intensity for plant biomass estimation differed slightly between years. In 2023, plant biomass was harvested from three replications in the infested field and one replication in the control field to maximize contrast under mite pressure during the initial screening phase. In 2024, biomass was harvested from three replications in both fields to provide a more balanced dataset for stability analysis across environments. Environments were defined as the combination of year and field condition for AMMI purposes, resulting in four test environments: Y1_A, Y1_B, Y2_A, and Y2_B. Above-ground plant biomass was harvested using a sickle, maintaining a consistent stubble height of 10 cm. Total fresh weight was measured immediately after harvesting using a Micro S-29 (Sasco Africa, Benoni, South Africa) weighing scale, and records were taken. Whole plant material (300 g) from each replication was oven-dried at 60 °C for 48 h in labeled oven bags for subsequent analysis. Total dry weight (TDW) was calculated from the oven-dried sub-samples.

### 2.2. Data Analysis

#### 2.2.1. Analysis of Variance and Statistical Model

Statistical analyses were performed using ANOVA with R software (2024.12.0) using the lmer package to evaluate the main effects and interactions using the following mixed model: Y_ijklm_ = μ + Y_i_ + F_i_ + R_k_(Y_i_ × F_j_) + B_l_(R_k_) + G_m_ + (G_m_ × Y_i_) + (G_m_ × F_j_) + ϵ_ijklm_, where Y_ijklm_ is the observed trait for the m-th genotype in the l-th incomplete block, within the k-th replicate, located in the j-th field and the i-th year; μ is the overall mean; Y_i_ is the fixed effect of year; F_i_ is the fixed effect of field i (infested and non-infested); R_k_(Y_i_ × F_j_) is the random effect of the k-th replicate nested within the specific field; B_l_(R_k_) is the random effect of the l-th incomplete block nested within the k-th replicate; G_m_ is the random effect of the m-th genotype; (G_m_ × Y_i_) is the random interaction effect between genotype and year; (G_m_ × F_j_) is the random interaction effect between genotype and field; and ϵ_ijklm_ is a residual error term.

Tukey’s Honestly Significant Difference (HSD) was used for post hoc analysis after ANOVA.

#### 2.2.2. Genetic Variability Parameters Estimation

PCV and GCV were calculated as:

$$GCV\;(\%)\;=\;\frac{\sqrt{\sigma^{2}g}}{x}\times 100,$$

where $\sigma^{2}g$ = genotypic variance, and

$$PCV(\%)=\frac{\sqrt{\sigma^{2}p}}{x}\times 100,$$

where $\sigma^{2}p$ = phenotypic variance, x = General mean [22].

Broad-sense heritability (H^2^) is defined as the ratio of the genetic variance component to the phenotypic variance component, expressed as:

$$H^{2}=\;\frac{\sigma^{2}g}{\sigma^{2}p},$$

where $\sigma^{2}g$ = genetic variance component, $\sigma^{2}p$ = phenotypic variance component [23].

#### 2.2.3. AMMI Analysis of Total Dry Weight

TDW was selected as the primary trait for AMMI analysis as it represents the integrated agronomic output and key yield component most relevant to forage production. The AMMI model was applied to partition the total variation into genotype, environment, and genotype × environment interaction effects. Principal Component Analysis (PCA) was performed on the interaction residuals to capture the major interaction patterns. The ASV and the WAASB were calculated for each genotype to quantify genotypic stability. Analyses were carried out in R software (version 4.4.1; R Foundation for Statistical Computing, Vienna, Austria) using the metan package [24].

ASV was calculated as [25]:

$$ASV\;=\;\sqrt{\left(\frac{SS_{IPCA1}}{SS_{IPCA2}}\right)\cdot {\left(IPCA1\;score\right)}^{2}+{\left(IPCA2\;score\right)}^{2}}$$

where *SS_IPCA1_* is the sum of squares explained by the first Interaction Principal Component Axis, *SS_IPCA2_* is the sum of squares explained by the second Interaction Principal Component Axis, the IPCA1 score is the genotype’s score on the first principal component axis from the AMMI analysis, and the IPCA2 score is the genotype’s score on the second principal component axis.

#### 2.2.4. Stress Tolerance Index

Genotypic performance under mite stress conditions was evaluated using the stress tolerance index (STI), computed as [26]:

$$STI=\frac{Ys\times Yp}{Y^{2}}$$

where Ys is the yield under stress conditions, Yp is the yield under non-stress conditions, and Y is the mean yield of all genotypes under non-stress conditions.

## 3. Results

### 3.1. ANOVA of Agro-Morphological Traits and Physiological Indicators Across Diverse Urochloa Genotypes

Analysis of variance revealed significant effects of spider mite infestation (field), genotype, and their interactions on PH, TN, NDVI, and VSI in *Urochloa* spp. (Table 3). For PH, TN, and VSI, significant differences were observed between fields (infested vs. non-infested), genotypes, year × genotype, and field × genotype interactions, with the infested field showing a marked difference with lower PH and TN and a higher VSI compared to the non-infested field. ANOVA for NDVI revealed a highly significant effect of field infestation by spider mites and a significant field × genotype interaction, suggesting differential genotypic responses under infestation. However, genotypic variation and year × genotype interaction were not significant (Table 3).

The ANOVA for total dry weight (TDW) showed significant effects of field, genotype, year × genotype, and field × genotype interaction, indicating that biomass yield was reduced by spider mite infestation, differed among genotypes, and varied in genotype response across years and field conditions (Table 4).

### 3.2. Estimation of Variance Components

Variance-component estimates differed among traits and between infested and control conditions (Table 5). Tiller number showed the highest phenotypic and genotypic coefficients of variation in both environments, whereas NDVI showed the lowest. Broad-sense heritability was high for tiller number, NDVI, and STI; moderate for plant height; and lower for TDW under infestation than under control conditions. For TDW, the extensive difference between PCV and GCV under infestation indicated a stronger environmental influence under mite pressure (Table 5).

### 3.3. AMMI Biplot Analysis for Stability and Adaptability of Urochloa Genotypes Under Spider Mite Infestation

**Figure 1:**
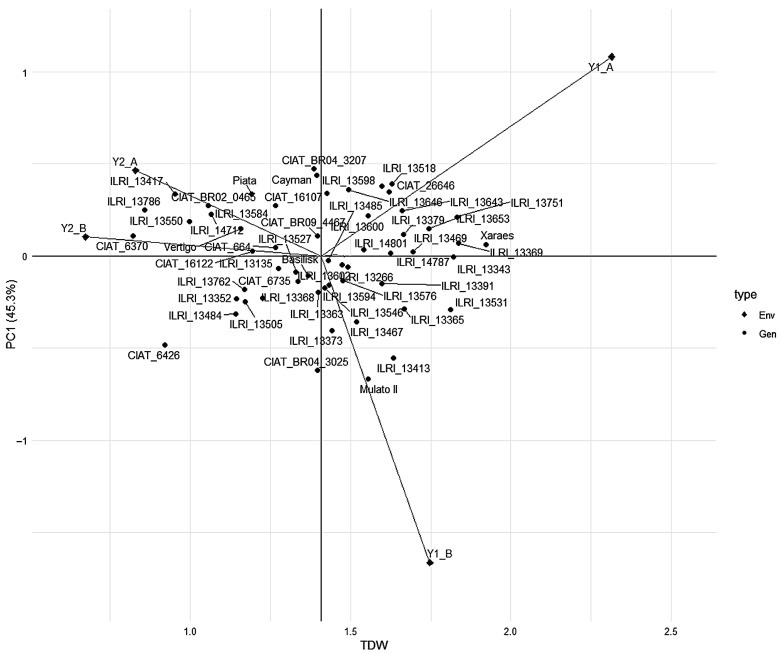
Additive main effects and multiplicative interaction (AMMI) biplot displaying *Urochloa* genotypes (Gen) and environment (Env) interactions across test environments for plant biomass yield (TDW).

The AMMI model for total dry weight (TDW) revealed significant genotype × environment interaction across the four environments, Y1_A, Y1_B, Y2_A, Y2_B, defined by year and field conditions (Figure 1). Genotypes positioned near the biplot origin had lower interaction effects and more stable TDW performance across environments. In contrast, those located farther from the origin displayed greater environmental sensitivity and more specific responses. The AMMI analysis thus highlighted differences in stability and consistency of TDW performance under contrasting spider mite conditions. Stability analysis ranking using the AMMI Stability Value (ASV) and the Weighted Average of Absolute Scores (WAASB) were conducted for total dry weight (TDW) of *Urochloa* genotypes (Supplementary Table S2). Based on the ASV and the WAASB, CIAT_16122, CIAT_664, ILRI_14801, ILRI_14787, and ILRI_13266 were the most stable genotypes, while CIAT_BR04_3207, Mulato II, CIAT_BR04_3025, ILRI_13751, and ILRI_13413 were the least stable.

### 3.4. Indices of Stress Tolerance on TDW in Various Genotypes Under Mite Infestation

**Figure 2:**
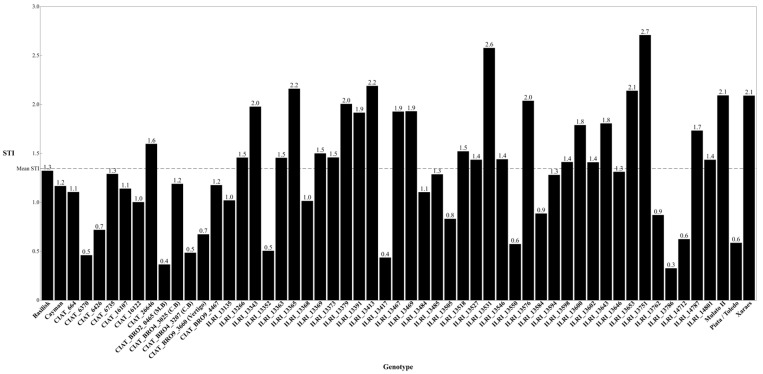
Stress tolerance index (STI) on total dry weight (TDW) of 55 *Urochloa* genotypes in two seasons under infested and non-infested conditions at *p* < 0.05.

The STI was estimated across a diverse panel of genotypes, displaying substantial yield variability in spider mite stress resilience. The overall mean STI was 1.34, serving as a baseline for comparative analysis. Genotypes ILRI_13751 and ILRI_13531 showed the highest STI values, at 2.71 and 2.58, respectively. In contrast, genotypes CIAT_BR02_0465 and ILRI_13786 exhibited the lowest STI values at 0.36 and 0.33, respectively (Figure 2).

## 4. Discussion

Mite feeding is known to disrupt photosynthetic capacity and assimilate partitioning, leading to stunted growth and reduced tillering [27]. In line with these reports, the present study showed significant effects of spider mite infestation on plant height (PH), tiller number (TN), and total dry weight (TDW), highlighting the considerable influence of pest pressure on the agronomic performance of *Urochloa* spp. [27]. Despite this, significant genotypic variation was observed for PH, TN, and TDW, indicating exploitable genetic variation within the germplasm. Such variation is essential for effective selection and sustained genetic gain in forage breeding programs [28]. In addition, PH and TN displayed high heritability across environments, confirming their reliability as a selection target regardless of pest pressure. However, TDW showed reduced heritability under infestation, reflecting increased environmental influence, a pattern consistent with reports that stress conditions increase environmental variance and reduce heritability estimates [13]. The presence of moderate to high heritability under stress indicates that genetic improvement for these key traits in forage production remains feasible when selection is conducted under representative stress environments. Tiller number emerged as a highly heritable trait under both infested and control conditions, reinforcing its importance as a reliable target for breeding stress-tolerant and high-yielding cultivars [29,30]. In perennial forage systems, the capacity of certain genotypes to maintain high tiller production under biotic stress is particularly advantageous, as it directly contributes to stand persistence, recovery, and long-term productivity [29].

The indices explored in this study proved valuable for characterizing the response of *Urochloa* genotypes to spider mite infestation under field conditions. The visual severity index (VSI) revealed strong field effects and significant genotypic variation, as well as year × genotype and field × genotype interactions, capturing both mite damage and differential genotypic responses to infestation. As such, the VSI emerges as a robust and pest-specific phenotyping tool for assessing host responses to spider mite infestation in *Urochloa* [31]. Similarly, the stress tolerance index (STI) was identified as a promising selection metric, displaying moderate heritability. This finding aligns with Fernandes et al. [32], who identified STI as a key indicator of stress resilience in food crops. Some *Urochloa* genotypes maintained high STI values under pest infestation, suggesting that breeding for stress tolerance is a viable strategy. Given the crucial role of forage crops in livestock farming, incorporating STI into multi-trait selection indices could strengthen breeding strategies aimed at stabilizing forage yields under increasing pest pressure [32,33]. In contrast to earlier expectations, NDVI exhibited high heritability despite low phenotypic variation, particularly under infestation, indicating strong genetic regulation of canopy greenness even when phenotypic differences were subtle. This phenomenon suggests that the NDVI, while limited in its ability to discriminate among genotypes under uniform conditions, may still serve as a useful indirect selection trait when combined with other agronomic and physiological measures. Although the NDVI is influenced by environmental conditions [34], its high heritability in this study highlights its potential value for monitoring stress responses. The presence of substantial genetic diversity within the studied germplasm suggests strong potential for breeding improved *Urochloa* genotypes tolerant to spider mites. Genetic variability is crucial for plant improvement, as it increases the likelihood of transgressive segregation, where novel and superior phenotypes emerge in breeding populations [35]. In this study, most traits assessed displayed greater variability under control conditions than under stress, consistent with Cortés [36], who reported that environmental stress limits phenotypic expression and reduces observable variation among genotypes.

The AMMI model provided an effective framework for interpreting genotype × environment interaction (GEI) in *Urochloa* biomass under contrasting mite-infested and non-infested conditions. AMMI distinguished stable TDW performance from environment-specific responses by partitioning additive and interaction components through ANOVA and PCA [37,38]. Complementary stability indices, the ASV and the WAASB, further refined genotype classification, consistent with their demonstrated utility in multi-environment trials of cereals and legumes [39,40]. The AMMI biplot showed that Xaraes, ILRI_13369, and ILRI_14787 combined high TDW with low interaction, making them strong candidates when both productivity and stability are prioritized. In contrast, CIAT_16122, CIAT_664, ILRI_14801, ILRI_14787, and ILRI_13266 had the lowest ASV and WAASB value, indicating broad adaptation and high stability across environments. Genotypes such as Basilisk, CIAT_BR09_4467, and ILRI_13485 were positioned near the origin and were stable, but their mean TDW was intermediate. Integration of the STI strengthened these findings by distinguishing broadly stable genotypes from stress-resilient genotypes. ILRI_13751 and ILRI_13531 exhibited a high STI under mite pressure. However, ILRI_13751 also ranked among the least stable based on ASV and WAASB, indicating specific adaptation to stress rather than broad stability. These results collectively highlight the value of combining AMMI-based interaction profiling, stability indices, and stress tolerance metrics to support cultivar selection and parental choice in breeding for spider mite-prone environments. Overall, combining the AMMI model, ASV, WAASB, and STI provides a selection framework by revealing a critical trade-off, enabling the identification of broadly adapted stable cultivars ideal for variable environments, and stress-resilient lines essential for zones with high pest pressure. This combination supports breeding decisions aimed at enhancing forage productivity and resilience under increasing climatic and pest pressures [5,41,42].

The observed reduction in biomass and vegetative growth under spider mite infestation indicates that mite pressure substantially affected forage performance, but the present study was not designed to resolve the underlying physiological or biochemical mechanisms of tolerance. Therefore, the identified differences among genotypes should be interpreted as phenotypic evidence of contrasting field responses. In addition, artificial infestation may not reflect natural pest dynamics, and indirect physiological effects from biweekly abamectin applications in control Field B cannot be completely excluded. Nonetheless, the design enabled robust estimation of genetic parameters (PCV, GCV, and H^2^), stability indices (ASV and WAASB), and stress tolerance (STI) under contrasting mite pressure.

## 5. Conclusions

This multi-season study revealed significant genotype, environment, and interaction effects, underscoring the importance of conducting comprehensive field studies in *Urochloa* breeding for spider mite tolerance. Traits such as plant height and tiller number were reliable selection traits, while TDW was more environmentally influenced under mite infestation. The high heritability of TN and STI confirmed their value for selecting stable, resilient genotypes under mite pressure. The NDVI, despite low variation, may serve as a complementary indirect selection trait. AMMI analysis identified Xaraes, ILRI_13369, and ILRI_14787 as high-yielding and stable genotypes. In contrast, the ASV and WAASB identified CIAT_16122, CIAT_664, ILRI_14801, ILRI_14787, and ILRI_13266 as the most stable and broadly adapted across environments. Basilisk, CIAT_BR09_4467, and ILRI_13485 were also stable, but showed intermediate TDW performance. STI revealed that ILRI_13751 and ILRI_13531 were stress-resilient under mite pressure, while CIAT_6426, ILRI_13352, and CIAT_BR02_0465 were highly susceptible to spider mites. These results highlight the potential of combining stability analysis, pest- and yield-specific indices, such as the VSI and the STI, and strategic breeding approaches, such as hybridization and gene pyramiding, to improve yield and stress tolerance. Identifying genotypes tolerant to spider mites provides a foundation for developing resilient, high-yielding forages that support East African livestock systems under biotic and climatic stress. Future studies should characterize breeding populations across different environments to develop high-yielding spider mite-tolerant interspecific *Urochloa* cultivars adapted to the East African context. Our next research phase will integrate trichome analysis and nutritional factor assessment to further elucidate resistance mechanisms and provide additional evidence for selecting superior genotypes.

## Figures and Tables

**Table 1:** Composition of the *Urochloa* diversity panel *.

Source	Type	Species	N
CIAT genebank	Accessions	*Urochloa decumbens*, *U. brizantha*	10
CIAT breeding program	Hybrids	*Urochloa ruziziensis* × *U. brizantha* × *U. decumbens*	7
ILRI genebank	Accessions	*Urochloa brizantha*	38
Total			55

* The full list of genotypes, species designations, and source institutions is provided in Supplementary Table S1.

**Table 2:** Visual severity scoring and confidence intervals.

Visual Severity Damage	% Damage	Damage
0	0%	No damage
1–3	10–30%	Moderately damaged
3–7	30–70%	Damaged—Visible chlorotic lesions
7–10	70–100%	Severely damaged—Insuperable chlorotic lesions

**Table 3:** Summary results of ANOVA evaluating variation in plant height, tiller number, NDVI, and visual severity index (VSI) of *Urochloa* genotypes (*N* = 55) under infested and non-infested (control) conditions in two years.

Trait	Source of Variation	SS	Mean Sq	DF	DenDF	F-Value	Pr(>F)	Signif.
Plant Height	Field	1439	1438.9	1	109.94	5.30	0.02	*
	Genotype	113,025	2093.1	54	831.46	7.72	<2.2 × 10^−16^	***
	Year × Genotype	50,768	940.2	54	834.95	3.47	1.55 × 10^−14^	***
	Field × Genotype	39,088	723.8	54	829.62	2.67	3.83 × 10^−9^	***
Tiller Number	Field	753,733	753,733	1	140.4	80.24	1.82 × 10^−15^	***
	Genotype	1,549,527	28,695	54	857.28	3.05	9.56 × 10^−12^	***
	Year × Genotype	893,287	16,542	54	844.62	1.76	0.0008	***
	Field × Genotype	748,725	13,865	54	856	1.48	0.016	*
NDVI	Field	0.59	0.59	1	139.66	101.40	<2 × 10^−16^	***
	Genotype	0.38	0.01	54	847.33	1.20	0.16	ns
	Year × Genotype	0.43	0.01	54	846.75	1.35	0.05	ns
	Field × Genotype	0.43	0.01	54	846.36	1.35	0.05	*
VSI	Field	769.26	769.26	1	125.33	462.41	<2.2 × 10^−16^	***
	Genotype	139.16	2.58	54	807.05	1.55	0.008	**
	Year × Genotype	202.06	3.74	54	846.85	2.25	1.55 × 10^−6^	***
	Field × Genotype	138.95	2.57	54	804.63	1.55	0.008	**

SS: Sum of Squares; Mean sq: Mean Square; DF: Degrees of Freedom; DenDF: Denominator Degrees of freedom; F-Value: F statistic; Pr(>F): *p*-value for the F-test; Signif.: Significant difference at *** *p* < 0.001, ** *p* < 0.01, * *p* < 0.05, ns—no significant difference.

**Table 4:** Summary results of analysis of variance and interaction effects on total dry weight (TDW) of *Urochloa* genotypes (*N* = 55) evaluated under mite and non-mite infestation across two years.

Trait	Source of variation	SS	Mean Sq	DF	DenDF	F-Value	Pr(>F)	Signif.
TDW	Field	6.59	6.59	1	56.85	18.88	5.808 × 10^−5^	***
	Genotype	23.8	0.44	54	277.43	1.26	0.01	**
	Year × Genotype	28.61	0.53	54	295.16	1.52	0.02	**
	Field × Genotype	25.2	0.47	54	277.32	1.34	0.04	**

SS: Sum of Squares; Mean sq: Mean Square; DF: Degrees of Freedom; DenDF: Denominator Degrees of freedom; F-Value: F statistic; Pr(>F): *p*-value for the F-test; Signif.: *** Significant difference at *p* < 0.001, ** *p* < 0.01.

**Table 5:** Estimates of variance components, PCV, GCV, broad sense heritability, and CV of agro-physiological traits of *Urochloa* (*N* = 55) genotypes in the years 2023 and 2024.

Traits	Field	Mean ± S.E	$\sigma^{2}p$	$\sigma^{2}g$	PCV%	GCV%	H^2^	CV%
TDW	Infested	2.24 ± 0.08	0.54	0.31	34.3	19.58	0.57	27.21
	Control	2.71 ± 0.08	0.41	0.4	33.19	32.03	0.98	34.22
PH	Infested	65.29 ± 2.13	317.56	211.31	25.69	17.11	0.67	24.16
	Control	75.03 ± 2.14	314.04	206.93	22.49	14.83	0.66	21.11
TN	Infested	187.41 ± 6.8	5677.54	4816.13	74.2	62.9	0.85	27
	Control	224.6 ± 8.55	11,224	9324.29	75.83	63	0.83	28
NDVI	Infested	0.77 ± 0.003	0.002	0.0018	1.62	1.3	0.9	3.05
	Control	0.73 ± 0.003	0.003	0.0023	1.81	1.57	0.77	5.79
**STI**		1.38 ± 0.04	0.05	0.04	21.8	16.51	0.8	22.41

TDW: Total Dry Weight (kg); PH: Plant height (cm); NDVI: Normalized Difference Vegetative Index; STI: Stress Tolerance Index; $\sigma^{2}p$: Phenotypic Variance; $\sigma^{2}g$: Genotypic Variance; PCV: Phenotypic Coefficient of Variation (%); GCV: Genotypic Coefficient of Variation (%); H^2^: Broad Sense Heritability; CV%: Coefficient of Variation.

## Data Availability

Passport data on accessions and information to request germplasm is available on https://www.genesys-pgr.org/ (accessed on 18 December 2025).

## Supplementary Materials

The following supporting information can be downloaded at: https://www.mdpi.com/article/10.3390/plants15071117/s1, Table S1: The full list of genotypes, species designations, and source institutions. Table S2: Stability analysis ranking using AMMI Stability Value (ASV) and Weighted Average of Absolute Scores (WAASB) for Total Dry Weight (TDW) of *Urochloa* genotypes.

## Abbreviations

C4	Carbon 4 Photosynthetic Pathway
CGIAR	Consultative Group on International Agricultural Research
CIAT	International Center for Tropical Agriculture (*Centro Internacional de Agricultura Tropical*)
HPR	Host-Plant Resistance
ILRI	International Livestock Research Institute
NDVI	Normalized Difference Vegetation Index
PH	Plant Height
TDW	Total Dry Weight
TN	Tiller Number
STI	Stress Tolerance Index
VSI	Visual Severity Index
GEI	Genotype by Environment Interaction
AMMI	Additive Main Effects and Multiplicative Interaction Model
ANOVA	Analysis of Variance
ASV	AMMI Stability Value
Den DF	Denominator Degrees of Freedom
DF	Degrees of Freedom
GCV	Genotypic Coefficient of Variation
H^2^	Heritability (broad-sense)
Mean Sq	Mean Square
PCV	Phenotypic Coefficient of Variation
Pr(>F)	Probability Value (from F-test)
Signif.	Significance Codes (e.g., *, **, ***)
SS	Sum of Squares
WAASB	Weighted Average of Absolute Scores from the Best Linear Unbiased Prediction
