# Supplementary material for: Spider Mite Response, Agronomic Performance, and Stability of a Urochloa spp. Diversity Panel Under Field Conditions
Source: Plants (Basel). 2026 Apr 5;15(7):1117. doi: 10.3390/plants15071117 (PMC13074608; doi:10.3390/plants15071117)
Supplement: Supplementary file 1 [file plants-15-01117-s001.zip › Supplementary Table S1_Genotypes.pdf]

Table S1. The full list of genotypes, species designations, and source institutions.

Accession number	Species
CIAT genebank	
**CIAT_664**	*Urochloa decumbens*
**CIAT_6426**	*Urochloa brizantha*
**CIAT_6735**	*Urochloa brizantha*
**CIAT_16107**	*Urochloa brizantha*
**CIAT_26646**	*Urochloa brizantha* cv. *MG4*
**CIAT_6370**	*Urochloa decumbens*
**CIAT_16122**	*Urochloa brizantha*
**CIAT_606**	*Urochloa decumbens* cv. *Basilisk*
**CIAT_16125**	*Urochloa brizantha* cv. *Piata*
**CIAT_26110**	*Urochloa. brizantha* cv. *Xaraes*
CIAT breeding program	
**CIAT_36087**	*Urochloa* hybrid cv. *Mulato II*
**CIAT_BR02_1752**	*Urochloa* hybrid cv. *Camello*
**CIAT_BR02_0465**	*Urochloa* hybrid cv. *Mestizo*
**CIAT_BR04_3025**	*Urochloa* hybrid cv. *Camello*
**CIAT_BR04_3207**	*Urochloa* hybrid cv. *Camello*
**CIAT_BR09_3660**	*Urochloa* hybrid
**CIAT_BR09_4467**	*Urochloa* hybrid cv. *Cayman*
**ILRI genebank**	
**ILRI_13266**	*Urochloa brizantha*
**ILRI_13135**	*Urochloa brizantha*
**ILRI_13786**	*Urochloa brizantha*
**ILRI_13646**	*Urochloa brizantha*
**ILRI_13391**	*Urochloa brizantha*
**ILRI_13531**	*Urochloa brizantha*
**ILRI_13352**	*Urochloa brizantha*
**ILRI_13484**	*Urochloa brizantha*
**ILRI_13643**	*Urochloa brizantha*
**ILRI_13485**	*Urochloa brizantha*
**ILRI_13343**	*Urochloa brizantha*
**ILRI_13505**	*Urochloa brizantha*
**ILRI_13467**	*Urochloa brizantha*
**ILRI_13600**	*Urochloa brizantha*
**ILRI_13602**	*Urochloa brizantha*
**ILRI_13417**	*Urochloa brizantha*
**ILRI_14801**	*Urochloa brizantha*
**ILRI_13598**	*Urochloa brizantha*
**ILRI_13469**	*Urochloa brizantha*
**ILRI_13584**	*Urochloa brizantha*
**ILRI_13365**	*Urochloa brizantha*
**ILRI_14787**	*Urochloa brizantha*

**ILRI_13527**	*Urochloa brizantha*
**ILRI_13373**	*Urochloa brizantha*
**ILRI_13368**	*Urochloa brizantha*
**ILRI_13751**	*Urochloa brizantha*
**ILRI_13413**	*Urochloa brizantha*
**ILRI_13546**	*Urochloa brizantha*
**ILRI_13594**	*Urochloa brizantha*
**ILRI_13379**	*Urochloa brizantha*
**ILRI_13363**	*Urochloa brizantha*
**ILRI_14712**	*Urochloa brizantha*
**ILRI_13369**	*Urochloa brizantha*
**ILRI_13653**	*Urochloa brizantha*
**ILRI_13762**	*Urochloa brizantha*
**ILRI_13576**	*Urochloa brizantha*
**ILRI_13550**	*Urochloa brizantha*
**ILRI_13518**	*Urochloa brizantha*

Urochloa hybrids are *Urochloa ruziziensis x U. brizantha x U. decumbens* from CIAT's breeding program.
