# Supplementary material for: Spider Mite Response, Agronomic Performance, and Stability of a Urochloa spp. Diversity Panel Under Field Conditions
Source: Plants (Basel). 2026 Apr 5;15(7):1117. doi: 10.3390/plants15071117 (PMC13074608; doi:10.3390/plants15071117)
Supplement: Supplementary file 1 [file plants-15-01117-s001.zip › Supplementary Table S2.pdf]

Supplementary Table S2: Stability analysis ranking using AMMI Stability Value (ASV) and Weighted Average of Absolute Scores (WAASB) for Total Dry Weight (TDW) of Urochloa genotypes.

GENOTYPE	Y	Y_R	ASTAB	ASTAB_R	ASI	ASI_SSI	ASV	MASV	MASV_SSI	SIPC	ZA_SSI	WAASB
CIAT_16122	1.19	42.00	0.12	29.00	0.01	43.00	0.03	0.03	43.00	0.03	70.00	0.02
CIAT_664	1.27	40.00	0.25	47.00	0.02	42.00	0.06	0.06	42.00	0.07	86.00	0.03
ILRI_14801	1.54	19.00	0.00	3.00	0.02	22.00	0.06	0.06	22.00	0.08	22.00	0.04
ILRI_14787	1.62	13.00	0.00	4.00	0.03	17.00	0.07	0.07	17.00	0.08	17.00	0.04
ILRI_13266	1.49	22.00	0.00	5.00	0.03	27.00	0.08	0.08	27.00	0.09	27.00	0.05
Xaraes	1.92	1.00	0.01	6.00	0.03	7.00	0.08	0.08	7.00	0.10	7.00	0.05
ILRI_13602	1.47	24.00	0.01	7.00	0.03	31.00	0.09	0.09	31.00	0.11	31.00	0.06
ILRI_13485	1.43	27.00	0.01	8.00	0.04	35.00	0.10	0.10	35.00	0.12	35.00	0.06
ILRI_13469	1.69	7.00	0.01	10.00	0.05	16.00	0.12	0.12	16.00	0.14	16.00	0.07
Basilisk	1.37	35.00	0.01	9.00	0.05	45.00	0.13	0.13	45.00	0.15	46.00	0.08
ILRI_13527	1.33	37.00	0.02	12.00	0.06	48.00	0.15	0.15	48.00	0.20	49.00	0.10
CIAT_6735	1.34	36.00	0.00	2.00	0.06	48.00	0.16	0.16	48.00	0.14	38.00	0.08
CIAT_BR09_4467	1.40	31.00	0.73	55.00	0.06	44.00	0.16	0.16	44.00	0.21	86.00	0.11
CIAT_6370	0.82	55.00	0.20	43.00	0.08	69.00	0.20	0.20	69.00	0.27	99.00	0.13
ILRI_13546	1.42	29.00	0.03	14.00	0.08	44.00	0.20	0.20	44.00	0.19	42.00	0.10
ILRI_13369	1.84	2.00	0.04	17.00	0.08	18.00	0.20	0.20	18.00	0.26	18.00	0.12
ILRI_13363	1.40	30.00	0.04	16.00	0.09	47.00	0.23	0.23	47.00	0.21	45.00	0.11
ILRI_13135	1.28	38.00	0.05	19.00	0.09	56.00	0.24	0.24	56.00	0.29	57.00	0.14
ILRI_13391	1.60	15.00	0.05	18.00	0.10	34.00	0.24	0.24	34.00	0.32	36.00	0.16
ILRI_13594	1.43	26.00	0.06	20.00	0.10	46.00	0.26	0.26	46.00	0.34	49.00	0.17
ILRI_13550	1.00	51.00	0.06	21.00	0.10	72.00	0.27	0.27	72.00	0.34	75.00	0.17
ILRI_13343	1.82	4.00	0.08	26.00	0.11	26.00	0.28	0.28	26.00	0.29	22.00	0.13
ILRI_13762	1.17	45.00	0.08	25.00	0.12	68.00	0.30	0.30	68.00	0.40	72.00	0.20
ILRI_13368	1.22	41.00	0.07	22.00	0.12	65.00	0.30	0.30	65.00	0.38	66.00	0.19
ILRI_14712	1.06	49.00	0.08	24.00	0.12	74.00	0.31	0.31	74.00	0.39	75.00	0.20
ILRI_13584	1.27	39.00	0.08	23.00	0.12	65.00	0.32	0.32	65.00	0.29	59.00	0.15
ILRI_13484	1.14	48.00	0.10	27.00	0.14	75.00	0.36	0.36	75.00	0.32	70.00	0.17
ILRI_13365	1.67	8.00	0.11	28.00	0.14	36.00	0.37	0.37	36.00	0.45	37.00	0.23
ILRI_13379	1.67	9.00	0.15	32.00	0.15	38.00	0.39	0.39	38.00	0.48	40.00	0.23
Vertigo	1.16	46.00	0.15	30.00	0.15	76.00	0.39	0.39	76.00	0.50	78.00	0.24
ILRI_13653	1.74	6.00	0.15	31.00	0.15	37.00	0.40	0.40	37.00	0.51	39.00	0.25

CIAT_16107	1.43	28.00	0.27	48.00	0.16	60.00	0.40	0.40	60.00	0.43	76.00	0.22
ILRI_13643	1.66	10.00	0.17	37.00	0.17	43.00	0.44	0.44	43.00	0.58	47.00	0.29
ILRI_13505	1.17	44.00	0.17	36.00	0.17	78.00	0.44	0.44	78.00	0.58	82.00	0.29
ILRI_13417	0.95	52.00	0.15	34.00	0.17	87.00	0.44	0.44	87.00	0.54	88.00	0.27
ILRI_13646	1.49	21.00	0.15	33.00	0.17	57.00	0.44	0.44	57.00	0.51	55.00	0.26
ILRI_13576	1.48	23.00	0.20	42.00	0.18	60.00	0.45	0.45	60.00	0.55	58.00	0.27
ILRI_13531	1.81	5.00	0.18	39.00	0.18	43.00	0.46	0.46	43.00	0.61	46.00	0.30
ILRI_13467	1.52	20.00	0.17	38.00	0.18	59.00	0.46	0.46	59.00	0.57	59.00	0.29
ILRI_13786	0.86	54.00	0.19	41.00	0.18	94.00	0.46	0.46	94.00	0.61	96.00	0.30
ILRI_13373	1.44	25.00	0.16	35.00	0.18	66.00	0.47	0.47	66.00	0.44	55.00	0.23
ILRI_13600	1.55	18.00	0.21	45.00	0.19	60.00	0.47	0.47	60.00	0.62	61.00	0.30
CIAT_26646	1.62	14.00	0.00	1.00	0.19	57.00	0.49	0.49	57.00	0.62	15.00	0.31
ILRI_13598	1.60	16.00	0.19	40.00	0.19	60.00	0.49	0.49	60.00	0.59	56.00	0.30
Piata	1.19	43.00	0.20	44.00	0.19	88.00	0.49	0.49	88.00	0.64	88.00	0.32
ILRI_13518	1.63	12.00	0.22	46.00	0.20	58.00	0.52	0.52	58.00	0.64	59.00	0.33
CIAT_6426	0.92	53.00	0.04	15.00	0.23	100.00	0.58	0.58	100.00	0.63	70.00	0.33
Cayman	1.40	33.00	0.02	13.00	0.23	81.00	0.58	0.58	81.00	0.72	47.00	0.37
CIAT_BR02_0465	1.06	50.00	0.02	11.00	0.24	99.00	0.62	0.62	99.00	0.81	60.00	0.40
ILRI_13352	1.14	47.00	0.41	51.00	0.26	97.00	0.65	0.65	97.00	0.83	98.00	0.40
ILRI_13413	1.63	11.00	0.35	49.00	0.26	62.00	0.67	0.67	62.00	0.77	60.00	0.40
ILRI_13751	1.83	3.00	0.49	54.00	0.28	55.00	0.71	0.71	55.00	0.88	55.00	0.42
CIAT_BR04_3025	1.40	32.00	0.36	50.00	0.30	85.00	0.76	0.76	85.00	0.87	82.00	0.45
Mulato II	1.55	17.00	0.49	53.00	0.31	71.00	0.80	0.80	71.00	0.88	71.00	0.46
CIAT_BR04_3207	1.39	34.00	0.45	52.00	0.35	89.00	0.90	0.90	89.00	1.18	87.00	0.58
